# Antiviral Inactivated Vaccines: Looking to the Past to Face the Future—A Narrative Review

**DOI:** 10.3390/vaccines13111140

**Published:** 2025-11-05

**Authors:** Francisca Hildemagna Guedes-da-Silva, Victor Augusto Roncaglia-Pereira, Sara Torres, María Camila Escobar García, Kelvinson Fernandes Viana, Jerson Lima Silva, Andréa Cheble Oliveira, Andre Marco Oliveira Gomes

**Affiliations:** 1 Institute of Medical Biochemistry Leopoldo de Meis and National Institute of Science and Technology for Structural Biology and Bioimaging, Federal University of Rio de Janeiro (UFRJ), Rio de Janeiro 21941-902, RJ, Brazil; hildemagnaguedes@gmail.com (F.H.G.-d.-S.); victorroncaglia@gmail.com (V.A.R.-P.); jerson@bioqmed.ufrj.br (J.L.S.); 2 Vaccine Development Technology Laboratory, Federal University for Latin American Integration (UNILA), Foz do Iguaçu 85870-650, PR, Brazil; sara.torres2307@gmail.com (S.T.); mce.garcia.2016@aluno.unila.edu.br (M.C.E.G.); kelvinson.viana@unila.edu.br (K.F.V.)

**Keywords:** inactivated vaccines, virus, immunizers, high hydrostatic pressure

## Abstract

Throughout human history, contagious infectious diseases have significantly impacted societies, shaping the fate of great dynasties and challenging economic and political systems, social relations, and the overall well-being of the human species. The SARS-CoV-2 pandemic brought unprecedented challenges, emerging in the context of extreme globalization and rapid technological development. The speed of viral spread, the highest absolute mortality rate caused by a viral agent in the last 100 years, and the severe economic and social consequences imposed an urgent need for vaccine development on a previously unimaginable timescale. The proven safety and efficacy of inactivated vaccines enabled the development and large-scale application of the first immunizer against SARS-CoV-2 in less than a year after the World Health Organization (WHO) declared the pandemic. In this review, we discuss the importance of inactivated antiviral vaccines and their historical impact in containing highly harmful diseases affecting humanity. We also explore the cellular mechanisms by which inactivated vaccines may induce immunogenic responses against viral pathogens. In addition, we bring to light a discussion about a fast, cost-effective, potentially efficient technology for large-scale immunizer production: High hydrostatic pressure (HHP), a method long supported by decades of preclinical studies and which is especially effective in the context of enveloped viruses. Finally, we discuss the role of inactivated antiviral vaccines in the face of advances in biotechnology and, therefore, the emergence of vaccines that use genetic engineering in their production, such as RNA, DNA and viral vaccines, which have gained special prominence during the COVID-19 pandemic.

## 1. Introduction

Inactivated vaccines have significantly contributed to global health by controlling infectious diseases due to their safety, stability, and cost-effectiveness. Despite the emergence of new vaccine technologies, traditional inactivated vaccines remain essential for their rapid and scalable production. Historically, the Salk poliomyelitis vaccine, enabled by groundbreaking poliovirus cultivation methods developed by Enders and colleagues, marked a major advancement in vaccine development [1]. The present review emphasizes High Hydrostatic Pressure (HHP) as an innovative virus inactivation method, preserving key immunogenic structures while effectively inactivating viruses such as influenza, yellow fever, and vesicular stomatitis virus. Comparatively, inactivated vaccines continue to demonstrate reliable safety and efficacy, especially important for rapid response in pandemic scenarios and resource-limited settings. Advances like HHP further ensure their ongoing relevance in global public health.

## 2. Pandemics/Endemics: Historical Background, the Development of Vaccines and the Impact on Global Health

**Figure 1:**
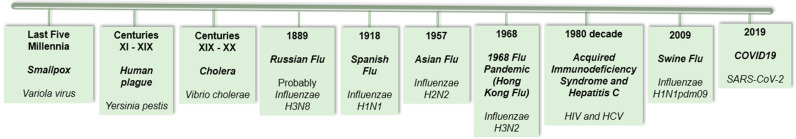
Remarkable endemic and pandemic events throughout history.

Infectious diseases have impacted humans throughout history (Figure 1). The expansion of urban spaces and trade routes has led to changes in natural ecosystems, increasing interaction between humans and fauna, and favoring the spread of infectious diseases. These factors have been crucial in the emergence of epidemic outbreaks on a global scale [2,3]. Historical records of epidemics and pandemics are closely associated with the decline of civilizations and empires [4]. *Yersinia pestis*, the etiological agent of the human plague, was responsible for approximately three pandemics between the 11th and 19th centuries. It played a fundamental role in the decline of at least two major empires—the Roman and the Byzantine empires—due to the high mortality rates [5,6].

At the beginning of the 19th century, the emergence of steamships and trains accelerated the process of globalization and enhanced communication between distant regions of the world. This development, combined with the lack of basic sanitation in urban centers, played a key role in the onset of seven cholera pandemics caused by the bacterium *Vibrio cholerae*, which affected the world throughout the 19th and 20th centuries [7,8].

Vaccines developed at the end of the 19th century to combat the spread of cholera, plague and typhoid fever were the first to use inactivated (killed) bacterial pathogens. These became the foundation for what would later evolve into inactivated viral antigen vaccines [9,10,11].

In this context, viral epidemics and pandemics have repeatedly reshaped human history (Figure 1), acting as catalysts for scientific innovation and global health policies. From smallpox and measles in ancient times to the devastating influenza pandemic of 1918, infectious viral diseases have caused immense mortality worldwide and social disruption, being related to major historical events, such as the beginning of the decline of the Roman Empire in an event known as the Antonine Plague [4,12]. These events underscored the urgent need for preventive measures and ultimately paved the way for the development of vaccines as one of the most effective tools in modern medicine.

In the context of vaccination, the smallpox virus played a pivotal role, with several key developments leading to the creation of the first vaccine. The earliest records of human passive immunization date back to Ancient Age—around 200 BCE—and describe the practice of variolation: the deliberate transfer of smallpox-infected material to healthy individuals to induce immunity [13,14].

The cornerstone of vaccination occurred at the end of the 18th century when the English doctor and scientist Edward Jenner observed that women who participated in cow milking were less susceptible to severe smallpox infections. He later extracted material from cow pustules and inoculated it into the arm of his gardener’s son, James Phillips. After exposing the boy to smallpox several times, Jenner noted that he did not become seriously ill or die. He presented his findings in the 1801 manuscript “On the Origin of Vaccine Inoculation”. Vaccination against smallpox led to one of the most important milestones in medical history: smallpox became the first infectious disease to be eradicated worldwide through vaccination [15]. This early intervention inspired the scientific foundations of immunology and preventive medicine. In the 19th century, Louis Pasteur expanded these principles through the development of vaccines based on attenuated or inactivated pathogens, establishing attenuation and inactivation as core strategies for inducing immune protection.

During the 20th century, rapid advances in virology and biotechnology led to an unprecedented expansion in vaccine development. The successful eradication of smallpox and the near-elimination of poliomyelitis illustrated the transformative impact of coordinated vaccination campaigns. Among the key technological milestones was the refinement of viral inactivation methods—initially through heat and formalin treatment—allowing the safe production of effective vaccines against poliovirus, influenza, rabies, and other pathogens. These inactivated vaccines provided robust safety profiles, could be manufactured at large scale, and were particularly suitable for mass immunization efforts in diverse populations.

Since the 19th century, major pandemics have been caused by viral agents that spread primarily through the respiratory tract, with notable exceptions such as the blood-borne pandemics of HIV and hepatitis C. The influenza virus, belonging to the genus *Alphainfluenzavirus*, has been responsible for recurrent pandemics from the 19th century to the present day [16]. The first well-documented influenza pandemic was the Russian flu, likely caused by the Influenza A/H3N8 virus [17]. Despite some reports citing a novel H3N2 virus as a possible etiological agent of the Russian flu pandemic [18,19], recent studies have suggested a potential coronavirus origin, particularly HCoV-OC43 [20,21]. However, this hypothesis remains under discussion, and no definitive evidence has been established. Following its emergence, the virus rapidly spread across the globe. In spite of its relatively low mortality rate, it claimed around 1 million lives between 1889 and 1900 [17,22].

Twenty five years after the onset of the Russian flu, an adapted subtype of an avian influenza virus, Influenza A subtype H1N1, was responsible for the Spanish flu, infecting approximately 500 million people and resulting in an estimated 50 million deaths worldwide [17,23]. In the century following the Spanish flu, several epidemics were caused by subtypes of the H1N1 virus, including the Asian flu, driven by the newly emerged H2N2 subtype, which killed around 2 million people in the 1950s [24,25]. A decade later, H3N2 was responsible for the 1968 flu pandemic, which began in Hong Kong. The new strain resulted from the addition of a novel H3 hemagglutinin from an avian virus and retained the N2 neuraminidase from the Asian flu virus. This pandemic killed over a million people and continues to circulate seasonally to this day [26,27].

Since its identification in the 1980s, Human Immunodeficiency Virus (HIV), the causative agent of Acquired Immune Deficiency Syndrome (AIDS), has driven a major global pandemic. To date, HIV has been responsible for approximately 44 million deaths, largely attributable to the HIV-1 subtype [28,29]. Despite significant advances in treatment, HIV remains a persistent pandemic, with approximately 40 million people currently living with the virus and more than 600,000 deaths from HIV-related illnesses annually [28]. Similarly, the hepatitis C virus (HCV) represents another major pandemic of a non-respiratory virus. Identified in 1989 [30], HCV is a blood-borne pathogen that has chronically infected more than 58 million people worldwide [31]. It is a leading cause of liver cirrhosis and hepatocellular carcinoma, resulting in approximately 290,000 deaths each year [31,32]. The parallel histories of HIV and HCV highlight that, while respiratory transmission is common for many pandemic viruses, it is not the exclusive route, as demonstrated by these impactful blood-borne pandemics. The “silent” nature of the infection—long periods of asymptomatic infection that masked its spread on a pandemic scale—is a key feature shared by both HCV and HIV, and is particularly defining for the natural history of Hepatitis C.

In 2009, a new subtype derived from H1N1 triggered the most recent major human influenza pandemic to date. The new etiological agent, Influenza A/H1N1pdm09, emerged through a triple reassortment involving the PB1 polymerase gene segments from human Influenza A H3N2, the PB2 and PA polymerase from avian influenza and the N1 segment from swine influenza [33]. Although this 2009 pandemic had a relatively limited impact, the ability of different types of influenza to undergo genetic reassortment and exchange gene segments still causes several outbreaks and maintains global surveillance for the potential emergence of future flu pandemics [34].

Despite their long-standing history, inactivated vaccines continue to play a central role in global immunization programs. Their safety, stability, and well-established production pipelines make them essential tools during emerging outbreaks and pandemics, when speed and scalability are critical. The most recent major pandemic began in late 2019, the COVID-19 pandemic, and renewed attention to these classical approaches, demonstrating that inactivated vaccine platforms remain a reliable and rapidly deployable solution, especially in resource-limited settings.

In COVID-19, the world was ravaged by the respiratory virus SARS-CoV-2, leading to a global health emergency declared by the World Health Organization (WHO) in March 2020. According to WHO epidemiological data, the COVID-19 pandemic officially infected approximately 800 million people and caused around 7 million deaths worldwide by 2024 [35]. In addition to the devastating effects on public health, SARS-CoV-2 brought about profound changes in lifestyle, causing significant changes in social interactions and impacting mental health.

In this context, the globalized lifestyle of the 21st century—with high population density, rapid international mobility, and the disruption of natural ecosystems due to industrialization—facilitates the transmission of infectious diseases through human contact, contributing to the dynamics of viral spread. Finally, it is important to consider the threat of future outbreaks caused by infectious agents that can threaten human life and disrupt socioeconomic stability. In anticipation of future outbreaks, the World Health Organization has proposed a scientific framework to prioritize potential pandemic pathogens and guide research preparedness [36]. Thus, in the face of a given state of public health emergency, the efficient development and provision of a vaccine in the short term is a global priority to minimize the damage caused by a pandemic.

Understanding the historical evolution and current applications of inactivated viral vaccines is crucial for guiding future preparedness strategies. By examining their scientific foundations, technological developments, and role in past and recent pandemics, valuable lessons can be drawn to optimize vaccine responses to future viral threats.

## 3. Classical Vaccines: Mitigation of Infectious Diseases

The first report of an inactivated antiviral vaccine dates back to the late 19th century, when Pasteur and colleagues developed a vaccine in 1885, using a partially inactivated rabies virus cultivated in rabbit spinal cord. This achievement was built upon early studies on inactivated (killed) bacterial vaccines for humans, targeting diseases such as typhoid fever, cholera, and plague [9,10]. However, the development of inactivated viral vaccines only advanced significantly with the advent of cell culture techniques. A major breakthrough occurred when John Enders, Thomas Weller, and Frederick Robbins discovered that poliovirus could be cultured in vitro using fibroblasts [1]—a finding that earned them the Nobel Prize in Physiology or Medicine in 1954. This innovation enabled the replication of viruses outside living organisms, paving the way for the large-scale production of viral vaccines.

The finding of Enders and colleagues enabled the development of the first inactivated virus vaccines against poliomyelitis. A landmark study published in 1953 by Jonas Edward Salk was pivotal in the history of inactivated virus vaccines [37,38]. Salk’s achievement extended beyond immunizing efficacy it demonstrated that vaccine development could proceed faster than usual: in only six years, the vaccine was developed, tested and initial data were released. Also, the development of Salk’s vaccine had the largest clinical trials at that point of the history with a cohort of over 1,300,000 volunteers [39]. These accomplishments make Salk’s inactivated vaccine one of the most successful examples of bench-to-bedside and rapid translational medicine in history.

Once Salk’s polio vaccines were declared safe and effective, the US federal agency responsible for licensing biological products recommended licensing to five pharmaceutical companies. Two weeks after Salk’s inactivated polio vaccines (IVP) were administered, some children became paralyzed. In all cases, the children had received the vaccine supplied by Cutter Laboratories. Subsequent studies showed that cellular debris prevented some particles from being adequately exposed to formaldehyde (the inactivating agent), preventing complete inactivation of the viral particles [39,40]. This case became known as the “Cutter Incident.” Today, modern IVP remains extremely useful for prophylaxis against paralytic polio. Since 2013, the WHO has reinforced the need to introduce at least one dose of IVP into vaccination programs to prevent vaccine-associated paralytic polio (VAPP) and circulating vaccine-derived poliovirus (cVDPV). This movement is called “The Plan” and is being developed by the Global Polio Eradication Initiative [41]. “The Plan” occurs in a context in which live attenuated Oral Poliovirus Vaccines (OPVs) have been implicated as the culprits of VAPP and cVDPV and have become a source of concern for the WHO in recent decades [42,43,44,45]. In the United States, OVPs were suspended and, since 2020, replaced by IPV [46]. VAPP and cVDPV reinforce the safety profile of IVP when compared to live attenuated OVP; however, the Cutter incident reinforced the need for extremely adequate quality control to ensure complete viral inactivation for vaccine production and highlighted the risks of a rapid transition between vaccine development and application.

Inactivated antiviral vaccines generally follow a similar production pathway: the virus is initially propagated using a substrate (such as primary cells, tissues, fertilized eggs, or even whole organisms) to generate large amount of viral particles [47,48]. These particles are then purified and concentrated before inactivation. The inactivation process can be performed using chemical, physical, or combined methods. Despite their toxicity, the first chemical agent used for viral inactivation with vaccine purpose was phenol, to produce an anti-rabies vaccine [49]. Since then, a wide range of inactivation methods has been described to successfully, including ascorbic acid [50], ethyleneimine derivatives [51], hydrogen peroxide [52], UV treatment [53] and Psoralen [54,55]. However, among all these, only the chemical agents, formaldehyde [56,57] and β-Propiolactone (BPL) [58,59] have been widely used for inactivation of licensed human viral vaccines.

Importantly, inactivation of a pathogen alone does not necessarily represent an effective vaccine; the preservation of immunogenic epitopes is crucial to induce protective immunity. Therefore, inactivation protocols must be carefully designed and validated to ensure both safety and immunogenicity.

### A Brief Comparative Overview of Live-Attenuated and Inactivated Vaccines

Live-attenuated and inactivated viral vaccines represent two of the most established and successful approaches in vaccinology, each with distinct advantages, limitations, and production challenges. Both aim to elicit protective immunity by exposing the host immune system to viral antigens; however, they differ fundamentally in how these antigens are presented and how the immune response is generated [11].

Live-attenuated vaccines are produced by weakening a virus so that it retains its ability to replicate in the host but loses its capacity to cause disease. Attenuation can be achieved through serial passage in non-human cells, targeted mutagenesis, or temperature-sensitive adaptations. Because they mimic natural infection, these vaccines stimulate robust and long-lasting immune responses, often with a single dose. They induce both humoral and cellular immunity, including strong mucosal responses when administered via oral or intranasal routes. Classical examples include the measles, mumps, and rubella (MMR) vaccine, the yellow fever 17D and 17DD vaccines, beyond the oral poliovirus vaccine (OPV).

However, the advantages of attenuated vaccines are counterbalanced by important safety and logistical concerns. The possibility of reversion to virulence, either through mutation or recombination, poses a risk, particularly in immunocompromised individuals. The OPV, for instance, has been associated with rare cases of vaccine-derived poliovirus (VDPV) [42]. Moreover, attenuated vaccines often require strict cold storage maintenance to preserve viral viability, complicating distribution in low-resource settings.

Inactivated vaccines, by contrast, are produced by physically or chemically destroying the infectivity of viral particles while preserving their antigenic structures. Formalin, β-propiolactone, or heat treatment are among the most commonly used inactivation methods. These vaccines are incapable of replication and therefore cannot revert to virulence, offering a superior safety profile [60]. As such, inactivated vaccines are considered safer for immunocompromised individuals [61]. The inactivated polio vaccine (IPV), influenza vaccines, and rabies vaccines are well-established examples. Inactivated vaccines are generally stable at higher temperatures than attenuated and are suitable for large-scale production and stockpiling.

The principal limitation of inactivated vaccines is their reduced immunogenicity. Because they do not replicate or induce intracellular antigen presentation, they tend to stimulate weaker cellular immune responses. Consequently, multiple doses or booster vaccinations are often required, and the inclusion of adjuvants is essential to enhance and sustain immunogenicity. Inactivated vaccines also elicit minimal mucosal immunity, which can limit their ability to prevent viral transmission [62].

From a manufacturing standpoint, inactivated vaccines can be developed more rapidly when high-containment facilities are available for the safe propagation of pathogenic viruses. Their production relies on standardized processes that have been optimized over decades, making them a practical choice during public health emergencies [63]. Conversely, live-attenuated vaccines demand more complex development pipelines and rigorous safety validation but can provide longer-lasting and broader immune protection once deployed [11].

In summary, live-attenuated and inactivated viral vaccines represent complementary strategies in the control of viral diseases. Attenuated vaccines excel in inducing strong and durable immunity but carry potential safety risks, while inactivated vaccines offer exceptional safety and scalability at the cost of reduced immunogenicity. The optimal choice between these approaches depends on the target pathogen, the epidemiological context, and the resources available for vaccine deployment. Ongoing advances in molecular attenuation, adjuvant design, and antigen stabilization are blurring the boundaries between these classical platforms, paving the way for next-generation vaccines that combine the best features of both.

Although modern vaccine platforms are currently available, it is worth noting that at the beginning of the 2020 pandemic, the first vaccine candidates approved for emergency use included inactivated vaccines against SARS-CoV-2, reinforcing the speed, low cost and efficacy of classical vaccine approaches [64]. In the case of the first licensed vaccine against SARS-CoV-2, the chemical agent chosen was β-propiolactone to inactivate the virus [58]. One key advantage of inactivated vaccines is their use of the entire virus as an immunogen, which elicits a broad antibody response against a variety of viral epitopes—in contrast to newer vaccine platforms that are based on specific epitopes, such as the SARS-CoV-2 Spike protein or partial protein fragments. On the other hand, systematic reviews and meta-analyses have shown that inactivated SARS-CoV-2 vaccines with β-propiolactone were less effective—and presented a more heterogeneous response—when compared to mRNA vaccines, which generally have efficacy of over 90% versus ~50% to ~70% efficacy of inactivated SARS-CoV-2 vaccines against confirmed infection and up to 82% against severe outcomes [58,59,60,61,64]. Conversely, Tanriover and colleagues reported in a large phase 3 study that the efficacy of the CoronaVac vaccine was over 83% in healthcare workers at the peak of the pandemic [65].

It is important to highlight that the overall rate of adverse reactions of inactivated vaccines in clinical trials is very low when compared to mRNA and adenoviral vector vaccines [66,67]. Chong and colleagues reported that two doses of SARS-CoV-2 inactivated vaccine booster after one dose of mRNA vaccine led to a neutralizing antibody response as high as individuals who receive two shots of mRNA vaccine avoiding adverse effects [68]. A comparison regarding the safety of inactivated vaccines and modern platforms will be discussed in Section 6.

The experience of immunization against Influenza presented some challenges that must be considering in the context of inactivated virus vaccines. The complex genetics of influenza viruses with high antigenic drift create an ongoing challenge in vaccine development to address dynamic influenza epidemiology. Although inactivated virus vaccines offer important advantages, such as safety and stability, experience with seasonal influenza vaccines highlights a crucial challenge related to the need for annual reformulation to keep pace with the rapid genetic evolution of circulating viruses [69,70]. The efficacy of these vaccines varies from year to year, depending on the degree of correspondence between the virus strains selected for vaccine production and the predominant circulating viruses in the population [71]. For instance, a new pre-pandemic inactivated vaccine it is been developed in Brazil to mitigate the potential risks of Influenza A H5N1, an etiological agent of Avian Flu [70].

In addition, inactivated vaccines are easier to store and distribute, making them highly suitable for low-resource settings [72,73]. While the advantages and limitations of modern vaccine platforms—including recombinant subunit, mRNA, and adenoviral vectors—are still under evaluation, the underlying production technologies, including the use of bioreactors and large-scale purification methods, are expected to be similarly applied across all vaccine types.

A successful vaccine must not only be safe and effective but also economically feasible and accessible, especially in developing countries where the burden of viral diseases is higher [48,56]. In addition to the above-mentioned aspects, for the success of an inactivated vaccine, the choice of adjuvants in vaccine formulations must be considered, observing their immunogenic potency, cost-effectiveness and safety. Thus, the successful development of new and improved adjuvants is key to future vaccine success, as they can enhance immune responses even at lower antigen doses, contributing to more affordable and environmentally sustainable solutions.

Currently, several types of adjuvants are available and the most common used in licensed or in clinical trial vaccines are the alum hydroxide (the first adjuvant ever used); oligonucleotide-based adjuvants (such as CpG and Poly I:C); oil-based adjuvants (for example, squalene-based formulations); and pathogen-associated molecular pattern (PAMP) such as monophosphoryl-lipid A [74,75]. To elicit a robust immune response, the inclusion of adjuvants is a determining factor in enhancing the immunogenicity of inactivated (or “killed”) viral antigens. Although adjuvants are considered, in general, very safe, some adverse effects of the vaccines such as narcolepsy, myofascitis and cardiovascular diseases are hypothesized to be related to the use of these adjuvants. However, the literature about this theme is poor; therefore, longitudinal studies are required to elucidate these potential aspect of vaccines adjuvants [76,77].

In addition, enveloped emerging RNA viruses are highly pathogenic due to their high mutation rate, resulting from the low fidelity of viral polymerases. This genetic variability promotes immune evasion and hinders the establishment of herd immunity. Inactivated vaccines preserve the structural integrity of the viral envelope proteins with conserved epitopes, inducing effective protective immunity with a favorable safety profile, high stability, low cost, and rapid development in endemic scenarios [61]. This highlighted the importance of inactivated vaccines in the face of the vast and threatening landscape of emerging infectious viruses found in nature. We believe that viral inactivation strategies may contribute to the development of effective and more affordable vaccines against emerging viruses.

## 4. Inactivated Vaccines: Potential to Induce Immune Response

**Figure 2:**
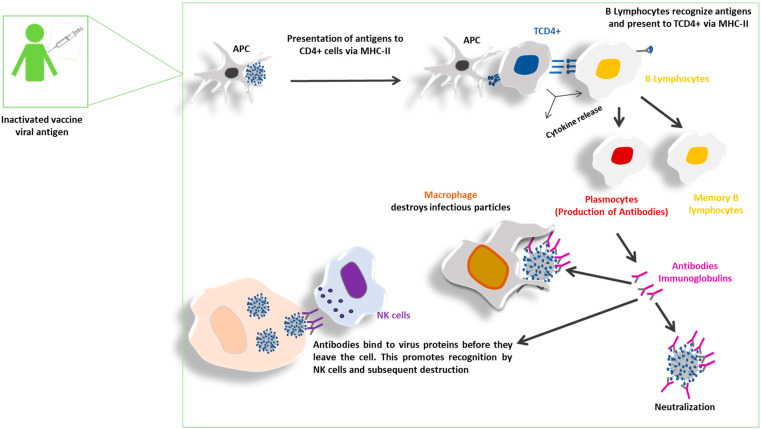
Effector mechanisms of the immune response induced by inactivated vaccines. Inactivated vaccines, produced from killed viruses, are recognized by antigen-presenting cells (APCs), such as dendritic cells, which process and present viral antigens via MHC class II molecules to naïve CD4^+^ T lymphocytes. This leads to the differentiation of helper T cells, which promote B cell activation and antibody production, mainly of the IgG isotype. These antibodies neutralize viral antigens and mediate opsonization, facilitating phagocytosis by macrophages or neutrophils. Although these vaccines elicit a strong humoral immune response, they often require adjuvants to enhance immunogenicity. The main effector mechanisms include antibody-mediated neutralization, complement activation, and Fc receptor–mediated phagocytosis.

In contrast to the innate immune system, the adaptive immune system—composed of B and T lymphocytes—is highly specific and has immunological memory mechanisms, enabling a faster and more effective response upon subsequent exposure to a given pathogen. B cells will produce antibodies in the humoral immune response, while T cells mediate the cellular response [78] (Figure 2).

Inactivated poliomyelitis vaccine also demonstrates great humoral seroconversion profile. Both Salk’s IPV and Sabin IPV displayed high seroconversion rates (>95%) for neutralizing antibodies after three high-dose administrations [79]. Inactivated vaccines against influenza—widely used since the 1980s—have shown seroconversion rates of up to 88% for Influenza A and 89% for Influenza B, as measured by hemagglutination assays in vaccinated individuals [80]. Antibody titers and hemagglutination inhibition data for H1N1 and H3N2 strains also revealed efficacy rates above 65% for both strains [81,82]. In the early 1990s, a formalin-inactivated hepatitis A vaccine was available, demonstrating a seroconversion rate of up to 95%, with antibodies persisting for at least 12 months [83].

It is well-established in the literature that inactivated antiviral vaccines typically induce a humoral immune response with the strong presence of specific neutralizing IgG antibodies, but only a weak cellular response mediated by T cells. Several studies in vaccinated animals, including non-human primates, using inactivated vaccines with different chemical agents (formalin and β-propiolactone) showed that these vaccines did not provoke an effective cellular immune response [58,59]. However, the study with the vaccine candidate BBV152 against SARS-CoV-2 revealed that immunized animals produced a cellular response profile [84].

Studies have shown that T cell responses in convalescent individuals are not long-lasting, with a significant decline observed approximately 10 months after natural infection. In this context, Ziwei Li et al. (2021) advocated for booster immunizations with inactivated vaccines to sustain immune efficacy and prolong CD4^+^ T cell responses [85]. These findings highlight that the specific T cell immunity induced by inactivated COVID-19 vaccines remains incompletely understood and requires further investigation. A study published by Yao Deng et al. (2021) reported that the inactivated vaccine BBIBP-CorV induced specific T cell responses that recognize multiple targets (proteins S, N, and E) of SARS-CoV-2 in 10 healthy individuals [86]. However, other groups have reported that the SARS-CoV-2-specific CD8^+^ T cell responses induced by inactivated vaccines were relatively weak and less frequently observed in vaccinated individuals compared to CD4^+^ T cell responses, supporting the idea that inactivated vaccines do not effectively stimulate strong CD8^+^ T cell responses [87]. Another key issue that must be addressed is the durability of humoral and cellular immunity elicited by inactivated vaccines.

The challenge remains in the hands of virologists and researchers in the field of vaccine development, in the ongoing search for applying classical or molecular technologies in the implementation of vaccines against emerging viruses, both in the public and private sectors. In this sense, it is necessary to consider alternative approaches, and new technologies should be encouraged, particularly when response to a vaccine is less than required or the vaccine becomes ineffective in the face of possible mutations that certain viruses may present.

## 5. High Hydrostatic Pressure (HHP): A Potential Platform for the Production of Inactivated Viral Vaccines

High hydrostatic pressure (HHP) has long been used in the food industry as a tool for food pascalization for preservation and sterilization. This method can reduce or avoid not only microorganisms but key harmful molecules as well [88]. Therefore, given the ongoing challenges in meeting global vaccine demands across diverse socioeconomic, demographic, and healthcare landscapes, HHP has emerged as a promising strategy for developing efficient, low-cost antiviral vaccines. This approach aims to induce robust immune responses using inactivated viral particles, without the need for toxic chemical inactivating agents [89].

Viral particles are macromolecular assemblies composed of proteins and nucleic acids, often surrounded by a lipid envelope. The structural integrity of these particles depends critically on protein–protein, protein–nucleic acid, and protein–lipid interactions. Among these, protein–lipid interactions are particularly susceptible to disruption by high pressure, although protein–protein and protein–nucleic acid interactions can also be affected. By perturbing these interactions, HHP can effectively inactivate viral particles while preserving essential immunogenic structures, making it a viable and safe platform for vaccine development [89,90].

In the literature, it is described that HHP induces, under pH-independent conditions, the fusogenic state of viral particles, which is, in general, an intermediate state of folding of the adhesion proteins of viral particles, essential for viral infection [91,92,93]. HHP can also mimic the conformational changes of virus adhesion proteins when they bind to the receptor on the cell membrane [94]. Other studies exploring the use of HHP in Picornavirus and evaluating the immunogenicity showed partial maintenance of the immune response [95,96], elicited neutralizing antibodies in rabbits [97] and showed high stability [98]. Interestingly, HHP has been shown to increase thermal resistance in certain attenuated poliovirus serotypes rather than leading to their inactivation. This unexpected outcome suggests a potential avenue for developing new strategies to improve heat stability of viral particles, which could be particularly valuable for improving vaccine storage and distribution [98].

Studies with the infectious bursal disease virus showed that the virus particles did not exhibit infectivity after 3 h at 1.8 kbar or after 2 h at 2.3 kbar, always at a temperature of 0 °C. Although intrinsic fluorescence spectroscopy analyses suggested structural changes in viral proteins, immunization with these inactivated particles generated protection in challenged chickens by the production of specific antibodies for the native virus [99]. It was also reported that the human rotavirus inactivated at 2.5 kbar for 60 min led to changes in the spike protein VP4, a hemagglutinin responsible for cell receptor binding and, the virus samples treated with high pressure presented high immunogenicity [94]. Similarly, vesicular stomatitis virus (VSV) presented a reduction of 4 Logs in infection titer after 12 h at 2.5 kbar and pressurized samples induced production of high concentrations of neutralizing antibodies even when compared with native particles [100,101,102].

Immunogenicity studies using high hydrostatic pressure (HHP) have also been conducted with various arboviruses. Yellow fever virus, a member of the *Flavivirus* genus and *Flaviviridae* family, was successfully inactivated at 3.1 kbar for 3 h at 4 °C. The pressure-inactivated virus conferred effective protection against both mortality and disease symptoms in mice [103]. Mayaro virus showed a 7-log reduction in infectious titer after 8 h at 2.5 kbar. Lipid fusion assays and transmission electron microscopy revealed that pressure induced fusion of viral lipid envelopes and irreversible alterations in the tertiary and quaternary structures of the envelope glycoproteins [92].

Among respiratory viruses, studies with influenza virus subtype H3N8 showed that the virus was inactivated after 12 h at 2.82 kbar and 25 °C, with a 7-log reduction in infectivity. H3N2 was inactivated after 3 h at 2.9 kbar. Both viruses present adequate immune responses and protection against infection [91,102]. Furthermore, intrinsic fluorescence spectroscopy and Bis-ANS fluorescence analyses showed that H3N2 was more sensitive to pressure due to the lower stability of its structural proteins’ quaternary structures [104]. Most recently, Brandolini and colleagues reported that SARS-CoV-2 B.1 and BQ.1.1 lineages were successfully inactivated and retained humoral immunogenicity when treated with 5 or 6 kbar for five minutes [105]. These data suggest that HHP induces changes in viral morphology, however, the structure of spike, membrane, and envelope proteins are retained.

Together, these findings highlight the potential of high hydrostatic pressure as a platform for vaccine development. The advantages of HHP are not only within the immunogenicity, but beyond this: it has a low cost, a high capacity of large-scale production, and fast development and offers the intrinsic safety of inactivated vaccines (Table 1). Despite its promise, High Hydrostatic Pressure (HHP) inactivation remains at an early experimental stage. To date, no HHP-based viral vaccine has been licensed for human use, and most available data derive from preclinical animal studies, which somewhat limits the amount of available data, making it difficult to compare it to other methods in terms of reproducibility, comparability, and translation of this technology to clinical use. Several challenges must be addressed before large-scale implementation: (i) reproducibility of antigenic structure following pressure treatment; (ii) standardization of pressure parameters across laboratories; (iii) validation of assays to monitor conformational integrity and residual infectivity; and (iv) manufacturing feasibility within biosafety-compliant facilities, since viral growth is still required prior to inactivation (Table 1). However, similarly, there was no mRNA-based vaccine approved for human use before the SARS-CoV-2 pandemic, and today this is one of the most promising technologies for the production of future vaccines. Nevertheless, the current literature points to HHP as a highly promising strategy for future vaccine development.

## 6. Safety: Inactivated Vaccines Versus Modern Platforms (Recombinant, mRNA, DNA and Vectored)

### 6.1. Inactivated and Recombinant Vaccines

Inactivated vaccines are produced from “killed” viruses, which makes them very safe, with a low risk of adverse effects. However, the induction of the immune response may be less robust and often requires booster doses to ensure long-term protection [106]. In the development and production of vaccines, the process of viral inactivation through chemical or physical methods prevents the phenomena of replication and mutation of these microorganisms, fundamental aspects of the pathogenic state.

Fine-tuning of the inactivation process should be considered, where excessive inactivation can nullify the immunogenicity of antigens, while insufficient inactivation may not produce an efficient immune response against the mechanisms of viral pathogenicity or even allow infectious particles to remain present, which can be disastrous, as in the case of the Cutter incident discussed above [107]. Inactivated vaccines are especially interesting for individuals with weakened immune systems, such as immunocompromised patients, the elderly, pregnant women, and lactating women, as they present important aspects that reinforce the safety profile of this type of vaccine, such as the absence of undesirable side effects and the risk of infection and transmissibility against the antigens used in the vaccine formulation [108,109].

Unlike traditional first-generation vaccines that use the entire inactivated and attenuated pathogen, second-generation vaccines developed from biotechnological advances use only specific parts of the pathogens such as purified proteins or toxoids. Recombinant vaccines produced through some protein peptides of viruses have a safety profile similar to inactivated vaccines, being generally well tolerated, with limited adverse reactions. However, despite being safe and targeted vaccines, the research and development process for these vaccine platforms is typically more time-consuming, as they require in-depth studies on viral structures, identification of specific cell receptors, and mechanisms of cell interaction [107,110]. Table 2 compares advantages and disadvantages between classical inactivated virus immunizers and third generation vaccines.

As with inactivated vaccines, recombinant vaccine platforms are widely recommended for vulnerable populations—including pregnant women, the elderly, and immunocompromised patients—due to their safety as they do not contain live attenuated biological agent. Studies have shown that inactivated influenza vaccines do not have adverse effects in pregnant women [111] and the elderly, protecting against viral infection with only mild side effects [112]. In children, inactivated influenza vaccines are both highly effective and rarely cause adverse events [113], while recombinant hepatitis B vaccines elicit robust immunogenicity, including in newborns [114].

Consequently, both inactivated and recombinant vaccines are indicated for individuals with chronic metabolic diseases such as diabetes and cardiovascular disease, reducing the risk of serious complications [115]. A study revealed that recombinant vaccines against herpes zoster also demonstrate high efficacy in individuals over 70 years of age, despite immunosenescence reducing the immune response in this populations [116].

### 6.2. mRNA and DNA Vaccines

Nucleic acids, DNA (deoxyribonucleic acid) and RNA (ribonucleic acid), are macromolecules that store and transmit genetic information of biological beings, playing crucial roles in protein synthesis and cell replication. Messenger RNA (mRNA) transmits information from the genetic code to the ribosomes from the DNA molecule, helping to identify the amino acid sequence required to synthesize specific proteins. Modern nucleic acid vaccine platforms are considered third-generation and can use fragments of mRNA from the virus to induce the production of specific proteins to activate the immune system, without presenting a potential risk of infection and genetic alterations, as the process occurs outside the cell nucleus and—in practical terms-do not require to grow up a large scale of pathogenic material [63]. The most common adverse effects in some individuals after administration of mRNA vaccines are mild and transient in nature [117].

mRNA vaccines have demonstrated excellent clinical outcomes, presenting a high safety profile comparable to that of clinical findings involving inactivated vaccines, although the safety history of inactivated vaccines is widely known due to their consolidated use over decades [118]. In this sense, both vaccines are effective in preventing infectious diseases and the choice between them depends on factors such as costs involving logistics, storage, availability and target population.

Limiting factors regarding mRNA vaccines are observed, since their development requires in-depth studies on the structures of the virus and its cellular interaction, high cost in the production process and instability of these immunizers due to the relative ease of degradation of nucleic acids when compared to classical vaccine models [119] Furthermore, clinical findings have associated mRNA vaccination against COVID-19 with low frequency cases of myocarditis, mainly affecting young men [120], and rare cases of autoimmune hepatitis after vaccination in women [121].

Possible mechanisms in the development of autoimmune diseases after this type of immunization include an anomalous immune response mediated by molecular mimicry and activation of autoreactive cells. However, further studies need to be conducted to elucidate these associations involving mRNA vaccines [121].

DNA vaccines are produced through a biotechnological process involving the production and purification of plasmids that synthesize copies of certain genes that will culminate in specific antigens. These vaccines have an important safety profile with a low risk of serious side effects, similar to the favorable effects of conventional vaccines [122,123]. During the COVID-19 pandemic, questions were raised about the mutagenicity of DNA vaccine platforms related to possible interactions between plasmids and the genome of the immunized individual. However, in safety studies of DNA vaccines against SARS-CoV-2, no interactions between plasmid DNA and mutagenic activity were observed [124]. In terms of immune response, DNA vaccines provide a specific response, being transcribed into RNA after entering the cell nucleus. Their efficacy is associated with the breakdown of the nuclear envelope during cell division, which allows the activation of both humoral and cellular immunity, essential to combat various pathogens, including emerging viruses [125].

Preclinical immunization studies in animals have not demonstrated undesirable effects related to pregnancy, fetal malformation, complications during childbirth and postnatal period. However, clinical studies need to be carried out to evaluate the potential effects of DNA vaccines in pregnant and lactating populations [109,126].

The effect of DNA vaccines on breastfeeding patients is also unknown [127], and it is necessary to investigate the interaction of DNA vaccines with breast milk production to understand whether there is excretion of any vaccine component in human milk. In 2022, the study developed by Abrignani et al. revealed hemodynamic disturbance of thrombosis as a rare adverse effect in immunized women [128].

Furthermore, information on the safety of these vaccine applications in immunosuppressed patients is still insufficient. However, the clinical study of the Indian DNA vaccine ZyCoV-D against SARS-CoV-2 indicated a safety profile for immunosuppressed patients consistent with the immunocompetent population [129]. In geriatric patients over 65 years of age, the ZyCoV-D vaccine showed obvious efficacy compared to applications in young patients and adults between 18 and 64 years of age, and there were no restrictions on this vaccine formulation in elderly patients [129,130]. Thus, the vaccine platforms have distinct characteristics, where ordinary inactivated vaccines with adjuvanted formulations induce a predominantly humoral immune response and require periodic booster doses [106] and DNA vaccines can induce humoral and cellular immunity without the need for additional adjuvants [131].

### 6.3. Viral Vector Vaccines

Viral vector vaccines provide genetic material from the pathogen that stimulates the humoral immune response and an effective cytotoxic cellular response, being more efficient in stimulating a robust immune response when compared to recombinant protein vaccines [132,133]. The viral vector vaccine platform is considered safe, however, in some rare cases, more serious adverse events of thrombosis and thrombocytopenia have been observed in immunized individuals, making a risk-benefit assessment necessary in patients with a history of thrombosis, thrombocytopenia and coagulopathies [134]. Furthermore, the pre-existing immunity against the vector could affect the vaccine efficacy [135].

Despite this, these vaccines have excellent stability, allowing their wide distribution in hard-to-reach regions [136]. The high level of safety of viral vector vaccines is guaranteed due to the genetic alteration of the viruses used, so that their pathogenicity is reduced or eliminated in the case of non-replicating viruses [133,137].

To date, there is insufficient data to ensure the safety and immunogenicity of viral vector vaccines, such as ChAdOx1, in children and adolescents under 18 years of age, and more clinical studies are required to better evaluate the outcome of immunization in this group [138]. Similarly, data in pregnant and lactating women are limited. Some cases of thrombosis with immune thrombocytopenia in women aged 32–54 years in the period 7 to 10 days after immunization with ChAdOx1/AstraZeneca and Ad26.CoV2.S/Janssen-Johnson & Johnson have been described [134,139]. These events are considered rare given the vast number of administered doses and the benefits of immunization outweigh the risks [140]. Thus, until further data are available, viral vector vaccines are not routinely recommended during pregnancy or breastfeeding, unless an individual risk-benefit assessment supports their use [132]. Hence, inactivated vaccines are considered safer for a general population due to the absence of viral replication, resulting in fewer adverse events [106]. While viral vector vaccines are effective, their safety profile may be more complex [62]. The choice of vaccine platform should consider the individual’s health status, vaccine availability, and the recommendations from public health authorities [141].

## 7. Conclusions

Inactivated vaccines have played an essential role in the history of vaccination and have greatly contributed to the control and elimination of numerous infectious diseases. For pandemic preparedness and outbreak response, first-generation vaccines based on inactivated pathogens still provide strategic benefits. Their enduring relevance is demonstrated by their strong safety profile, scalable production using current manufacturing infrastructure, and fit for low-resource settings. In particular, innovative inactivation methods such as high hydrostatic pressure may offer promising advancements for this platform. HHP-based inactivation can enhance immunogenicity while preserving safety and low manufacturing costs by allowing antigen preservation without the use of chemical agents or high temperatures. Inactivated vaccines remain a crucial and practical tool for bridging the gap between the onset of an outbreak and the introduction of more sophisticated, second- or third-generation platforms, especially in resource-constrained environments. However, the timely development of these vaccines relies on rapid access to viral isolates and supportive public policies that facilitate the swift mobilization of production capabilities. Building a flexible and robust immunization strategy capable of addressing future challenges in virology and global health depends on supporting both conventional inactivated vaccines and next-generation vaccine platforms.

## Figures and Tables

**Table 1:** HHP compared to the most used production methods for inactivated vaccines: pros and cons.

Method	Mechanism	Advantages	Limitations/Risks
Formaldehyde inactivation	Covalently cross-links viral proteins and nucleic acids, blocking replication.	Long-established and well-characterized method. Broadly applicable to many viruses. Simple equipment requirements. Regulatory precedent for human vaccines (e.g., IPV, HAV, rabies).	Slow process (days to weeks). May alter conformational epitopes, reducing neutralizing antibody responses. Requires removal of residual formaldehyde. Reproducibility depends on precise control of pH, temperature, and time.
β-propiolactone (BPL)	Alkylates nucleic acids and some amino acid residues, rapidly inactivating viral genomes.	Faster than formaldehyde (hours). Effective for enveloped and non-enveloped viruses. Widely used in influenza and COVID-19 inactivated vaccines. Hydrolyzes spontaneously into non-toxic byproducts.	Can modify antigenic sites if conditions not optimized. Requires strict temperature control. May leave residual BPL adducts if not fully hydrolyzed. Potential protein aggregation affecting potency.
High Hydrostatic Pressure (HHP)	Disrupts non-covalent interactions in viral capsid/envelope, preserving many structural epitopes.	No chemical residues. Scalable and environmentally friendly. Preserves antigenic structures better than heat or chemicals (in some viruses). Potential for rapid, uniform inactivation.	Still experimental (no licensed HHP-based human vaccine). Requires virus cultivation prior to inactivation (biosafety remains needed). Reproducibility and standardization not yet validated. Equipment cost and pressure uniformity are technical challenges.

**Table 2:** Comparative analysis of vaccine platforms highlighting key biological, technological, and clinical aspects.

Vaccine	Definition	Security	Infection	Adverse Effect	Adjuvant	Stability	Immunogenicity	Vulnerability
**Inactivated**	Inactivated pathogen	Mild reactions	Null, pathogens are dead and do not replicate	Local reactions, mild fever, fatigue and muscle pain	Requires adjuvants	High stability, simple storage (2–8 °C)	Need of booster doses	Safe for vulnerable groups
**RNA**	Messenger RNA that encodes viral proteins	Adverse events are rare	Null, RNA is rapidly degraded	Fever, fatigue, local pain, headache, mild lymphadenopathy	Require adjuvants	Lower stability; requires deep freezing (−70 °C)	Superior to inactivated and viral vector. Need of booster doses	Ongoing studies in vulnerable groups
**DNA**	DNA plasmids that encode viral antigens	Discussion about risks of integration into genome	Null, plasmid DNA does not cause infection	Local reactions, mild fever, fatigue	Requires or not adjuvants	Greater thermal stability than RNA (−70 °C)	Relatively low	Deficiencies in vulnerable groups
**Viral Vector**	Non-pathogenic virus used to deliver genetic material	Possible response to the viral vector	Null in non-replicative vectors	Local reactions, fever, fatigue, thrombocytopenia in severe cases	Does not require adjuvants	High stability, simple storage (2–8 °C)	High without the need for adjuvants	Evaluated for prior immunity to the vector
**Recombinant**	Purified viral proteins produced by genetic engineering	Low risk of adverse events	Null, proteins are not infectious	Mild reactions, local pain, fatigue, mild fever	Requires adjuvants	High stability, simple storage (2–8 °C)	Elevated if accompanied by adjuvant	Generally safe for vulnerable groups

## Data Availability

No new data were generated for this study.

## Abbreviations

The following abbreviations are used in this manuscript:

HHP	High Hydrostatic Pressure
WHO	World Health Organization
COVID-19	Coronavirus Disease 2019
HIV	human immunodeficiency virus
HCV	Hepatitis C virus
IPV	Inactivated Poliomyelitis Vaccine
OPV	Oral Poliomyelitis Vaccine
(VAPP)	vaccine-associated paralytic polio
(cVDPV)	circulating vaccine-derived poliovirus
BPL	β-Propiolactone
APCs	Antigen-Presenting Cells
MHC	Major Histocompatibility Complex
Th1	T helper 1
Th2	T helper 2
IgG	Immunoglobulin G
FMDV	Foot-and-Mouth Disease Virus
VSV	Vesicular Stomatitis Virus
CpG	Cytosine-phosphate-Guanine
Poly I:C	Polyinosinic:polycytidylic Acid
PAMP	Pathogen-Associated Molecular Pattern
Fc	Fragment crystallizable (antibody region)
Bis-ANS	4,4′-Dianilino-1,1′-binaphthyl-5,5′-disulfonic Acid
ChAdOx1	Chimpanzee Adenovirus Oxford 1
Ad26.CoV2.S	Adenovirus serotype 26-vectored COVID-19 vaccine
